# Chicago Medical Society

**Published:** 1883-07

**Authors:** 


### ARTICLE XI.

### CHICAGO MEDICAL SOCIETY.

The society held a regular meeting May 7, 1883, at the Grand Pacific Hotel. Dr. D. W. Graham presided. The minutes are somewhat extensive, but the following embraces the principal portion of the report:

Drs. Robert Randall and James E. Henderson were elected to full membership by the secretary (upon vote) casting the affirmative vote of the society.

Dr. E. F. Ingals stated the salient points contained in his paper on "The Treatment of Empyema," read at the previous meeting. He thought a rubber tube introduced was sufficient for drainage purposes; that a double trocar should be used to perforate the walls of the chest.

Dr. J. A. Robinson inquired how he introduced the trocar, and before taking his seat, Dr. R. stated he thought there would be contraction of the ribs which would render it difficult to retain the drainage tubes *in situ.* He believed it was absolutely necessary to keep the pleural cavity washed out as clean as possible, for occlusion was apt to occur.

Dr. R. Park received a suggestion from Prof. Nussbaum that glycerine was the best remedy used to fill the cavity. He (Dr. Park) thought if such was the case, that boro-glycerine was a still better remedy to use in these cases, introduced through the drainage tube. He recited a case of destructive empyema, where the resection of three ribs was necessitated. The case progressed favorably for some time, until a further extended necrosis of the ribs occurred, and the patient died. He believed it was the universal opinion that it was necessary to keep the cavity well cleansed.

Dr. Ingals closed the discussion by answering Dr. Robinson's question relative to the introducing the trocar, saying that the perforation was done between the ribs; he had never yet wounded an artery, and the pleura would readily heal. He thought glyeerine was an excellent remedy to use, especially in multilocular pleurisy. These cases nearly all die—however, a case of this kind may possibly recover. If occlusion was threatened early, keep in a silver canula to prevent it, but the drainage tubes of rubber are perfectly tight for ten or eleven days, before they are crowded out. He was sorry to say that it was not the unanimous opinion to keep the cavity washed out.

Dr. Frank S. Johnson exhibited Dr. Wm. P. Verity's new derrick apparatus, and explained its mechanism and advantages in the treatment of spinal difficulties and applying plaster casts, compared with Prof. Lewis Sayres' method Some of its advantages consisted in its cheapness and portability, and lightness in weight, doing away with all buckles, and strong, dark brown woolen cloths were substituted for leather, being equally as durable, and walnut, cedar or mahogany wood is used instead of the iron cross-bar above the head. A demonstration of lifting a boy sixteen years old, and suspending him a few moments, with no pain, proved the easy manner and facility with which it was accomplished, and mode of appliance in the treatment of curvatures of the spine.

Dr. Ephraim Ingals gave some observations on the published proceedings of the Illinois State Board of Health, regarding its meeting of April 12-14, 1883, in this city. Several sections of the report were read and elaborated upon, some of the remarks being as follows:

Relative to the Joplin Medical College, of Joplin, Mo., he had never heard of it before, but his recent researches informed him that there was a town in Missouri by that name, containing 7,000 inhabitants, and two medical colleges. He would not criticise the action of the Board in determining to recognize the diplomas of these colleges—and the same is true regarding the Indiana Eclectic Medical College, of Indianapolis—but in a town of the size of Joplin, he conld not see how the colleges there were supplied with sufficient subjects for demonstrating purposes.

Regarding the 3,800 non-graduates in this State in 1879—the year the Medical Practice Act went into effect—who were practicing medicine, he thought 1,000 was nearer the number; while the State now contains about 650 non-graduates. Physicians are increasing in greater ratio than the population, and poor orthography in letter-writing, and illiteracy, were too prominent features in many of our recent graduates. He now desired to present for discussion the following resolution:

*Resolved*, That the public good would be promoted by the establishment of a State Board of Medical Examiners; such board to be entirely separate from and independent of all medical colleges, to have the exclusive right to grant license to practice medicine in the State of Illinois, leaving to medical colleges their function of teaching and conferring degrees, but obliging all who in future desire to enter upon practice, and who have not already received license to do so, to go before such board to prove their fitness; and that said board be required carefully to examine all applicants as to their moral, literary and medical attainments, and only to confer a license on those who are well qualified in all these respects.

In presenting the resolution, the author of it said he never was clearly in favor of it, but had no doubt that its passage would be beneficial.

In the discussion, Dr. J. G. Kiernan said he had been a medical journalist for some time, and in that capacity was obliged to revise a large number of communications from physicians in New York, Philadelphia, and a few from Chicago. Many times the spelling was poor, and he gave an instance of how a New York graduate spelled the word emulsion. He thought students graduated too hurriedly, and he therefore favored the resolution.

Dr. J. H. Hollister stated that some phases of this subject had interested him for years. Improvement, however, is being advanced gradually in educating students. With reference to elevating a student's knowledge to a higher degree before entering college, he was personally interested, but thought there should be some common standard by which students in all the medical colleges in the State should be measured, and examined by several medical bodies. He thought an examining board might be selected from the Illinois State Medical Society that would satisfy the colleges, or examiners appointed by the Governor, even though it be centered in political interest, might form the best examining board; but the appointment should be given to those who are faithful to their profession, and their "standard of measurement" should be respected, and the appointees constituting this board should consider it an honor of great value. This doubtless would excel all others. The colleges, he thought, could with propriety delegate to this board what they should do, for it might be possible for a State Board to require strict junctures, so that students may become afraid to enter colleges here, and go to colleges in the cities of other States. He was aware that "phonetics" in orthography prevailed to some extent, but even those who were thus deficient might be well posted, and competent to practice. He favored a resolution looking toward a common center by an individual board.

Dr. R. E. Starkweather spoke independently of any college and of the State Board, but thought 3,800 was virtually the correct number of non-graduates that were in the State in 1877. Thirty per cent. of this number have since attended medical colleges and received their diplomas. He used to be associated with the State Board, and had no doubt that 75 to 100 very badly spelled letters were in their possession, not unlike the one published in their proceeedings of April 12.

He thought a doctor could not collect his fees (legally speaking) unless he had passed the examination of the State Board. We should all conform to the Medical Practice Act. He believed that preliminary examination of students should be carried out here—and elsewhere—by a State Board, and thought the colleges should graduate a less number than they do. Our colleges do not compare with some of those in Europe or Canada. He hoped this State would be the pioneer in the preliminary examination. We cannot have too few medical colleges. We should endorse the resolution, or encourage the State Board. Really, the latter body may not survive long, as the appropriations for defraying its expenses, according to newspaper reports, are liable to be cut short, or dispensed with entirely.

Dr. Kiernan said it was our duty to notify the State's Attorney and State Board of irregularities in the profession, that those doing so may be subject to penalty.

Dr. Simon Strausser related several amusing illustrations concerning M.D.'s. Among those cited was the following: His wife had a dressmaker who was rather bright. One day she bade them goodby, saying, "I am going to be a doctor." "Little over a year afterward, my wife and I received an invitation from the former dressmaker to go and see her graduate. We went, and sure enough, there she was, with a white rose in her ear, and she was given her diploma. I asked her if she had practiced dissections? She replied, 'Oh, we don't have dissections in our college.' However, a short time subsequently she married, as practice was not very good."

Two years ago, a man came to him and asked to enter into partnership, saying that he had been associated with a regular physician as salesman, and received half the profits derived from selling a patent medicine. Dr. Strausser denounced him, and last year this same man was made a full-fledged physician by securing a diploma from a college. The new doctor remarked that his diploma was paid for, too. Other cases were cited, showing how easy it was to enter the medical profession by securing a diploma.

So far as the State Board was concerned, he endorsed it, and thought a higher standard than the present one should be established.

Dr. C. W. Purdy, graduate of the Queen's University, Ontario, Canada, spoke of the merits a man must possess before graduating there. Each Territorial Division in Canada has a man who constitutes a medical council, that appoints an examining medical board. A student passes this board before entering a medical college in Ontario, and there is obliged to study four (4) years before graduating. This was not the case in America. He favored a higher degree of literary attainment, also the State Board or this resolution.

Dr. R. Park spoke on the legal status of the subject, and said every physician should become a licentiate, as he doubted if a physician could collect a bill in this city or State, unless licensed by the State Board to practice. The colleges were not thorough enough here. He thinks it requires ten years' study in Sweden before a student may graduate there. He doubted if our State would ever support a medical department to a university, and thought that the State Board should be sustained. He had heard the remedy Tarrant's "Excelsior Aperient" pronounced in this hall, by practical medical men, showing how unfamiliar they were with one of the simplest remedies.

Dr. G. C. Paoli had practiced in Copenhagen before coming to this country some thirty-four years ago. The venerable doctor said that many years ago a student there had to pass several degrees before graduating. Our colleges here were not particular enough, and sent out too many graduates in too short a time.

Dr. Kiernan moved that the resolution be laid on the table temporarily, not seconded.

Dr. J. H. Etheridge said the resolution was not properly before the society, as it had not been seconded, and the discussion so far was not pertinent to it, and was a criticism on the State Board of Health.

Dr. Starkweather arose to second the resolution, and Dr. Ingals answered some minor points not stated herein, when a vote was taken on passing the resolutiou, which was unanimously carried and adopted.

Dr. Ingals also offered the following:

*Resolved*, That a committee of three be appointed by the chair to represent the Chicago Medical Society, and that they be instructed to confer with the Illinois State Board of Health, relating to statements contained in the proceedings of its last meeting, and that this society respectfully requests said board to communicate to our committee any facts in the possession of the board that will enable the committee to prepare its report for the society.

Dr. E. H. Thurston thought this resolution should also pass—he would endorse it.

Dr. Starkweather seconded this second resolution just read by the venerable doctor, and upon vote, it too was unanimously adopted, showing how well indeed their value and merits were appreciated.

The chair announced that he would name the committee at the next meeting.

The following additional delegates to the State Society were appointed: Drs. S. J. Jones; J. H. Hollister; N. S. Davis; R. Park; D. W. Graham; J. E. Best; A. H. Foster; Edmund Andrews; A. R. Reynolds; L. H. Montgomery.

Thirty-five members and three visitors were present. Adjourned for two weeks

Among the 35 members present were Graham, Holmes, Bogue, Hollister, Etheridge, E. Ingals, E. F. Ingals, Strausser, Allport, Jones, Robison, Bidwell, Starkweather, Kiernan, Randall, Todd, Stevenson, D. H. Montgomery, Quine, Coey, Walton, Reynolds, Paoli, Purdy, Gray, D. M. Tucker, Lagorio, F. S. Johnson, Thurston, W. H. Curtis, Goldspohn, Park, and three others.

Visitors, C. G. Wheeler and two others.

L. H. MONTGOMERY, *Secretary.*

---

THE CHICAGO MEDICAL SOCIETY.

This society held its regular bi-monthly meeting May 21st, with Dr. D. W. Graham in the President's chair.

Several new petitions for membership were read, and Dr. Homer M. Thomas was unanimously elected a member of the society.

Dr. John E. Owens read a valuable and condensed report on "Recent Research in Operative Surgery." The first case cited was "Nephrectomy for Scrofulous Kidney." The history —the emaciated and general cachectic condition of the patient, combined with pyuria, and other circumscribed symptoms,— pointed to a scrofulous pyelitis of the right side. Medicines in these cases are inefficient. Soon after the operation, extreme collapse supervened, from which the patient never rallied. In this case a portion of the twelfth rib was excised. This class of cases requires close study in order to determine if by operation the whole disease can be removed, and the relative merits of nephrotomy ane nephrectomy, or if merely incising the kidney will give a chance for life. (This case was first read at a meeting of the Clinical Society, of London).

*Digital Exploration of the Bladder through incision of the Urethra from the Perinœum.*—This median operation has excited much interest, and when the exploration is made the bladder must be empty, then by concerted movements of supra-pubic pressure with the right hand, and slight movements of the left index in the bladder, almost every portion of the internal coat of the bladder may be brought under examination. (A case is reported in the London *Lancet*, June, 1882) where a fibrous tumor the size of a chestnut, coated with phosphatic matter, was diagnosed by digital exploration, and removed with a small pair of forceps, followed by rapid recovery. The procedure of Sir Henry Thompson is not a cystotomy, as the incision is only made into the urethra and a very little way into the prostate.

*Colectomy* (a short and available designation applicable to the cutting out, resection or excision of any part of the colon, thus distinguishing it from colotomy).—The subject of the clinical lecture from which this extract is taken was a case of chronic intestinal obstruction, the seat and cause of which could not be ascertained, but which was discovered on median abdominal section to be due to a circumscribed cylindrical growth, situated in the descending colon. (Case reported by Dr. Jas. Marshall, London *Lancet*, July, 1882.) An inch of the intestine was removed, together with the growth, but the patient only lived about two and a half days. The autopsy revealed diffuse peritonitis, starting from the lumbar wound. Very few cases of alleged attempted or complete colectomy for cancer of the large intestine are recorded. Of seven cases reported, from which we quote, four may claim to have prolonged life many months, while of the three remaining, they each proved fatal in fifteen hours to seven days. A single abdominal incision is an element of success in this operation.

*Wounds of the Heart.*—Black has recently maintained that wounds of the heart should receive surgical treatment. He pointed out, at the German Surgical Society, that death from wounds of the heart is usually due to asphyxia, in consequence of (London *Lancet*, March 10, 1883) hæmorrhage into the pericardium, to loss of blood, to damage to the motor ganglia of the heart, or to obliteration of the coronary artery. Hesitation in opening the thoracic cavity leads the surgeon to allow the patient to die from asphyxia, when he might be saved by a single incision into the pericardium. He experimented on four dogs where the pleural cavities, the pericardium and apex of the heart were opened for a short time, and all survived. [Wounds of the heart are fatal in so short a space of time that death results before the arrival of a surgeon to operate in the method suggested in the paper.—L. H. M.]

*The Removal of a Tumor from the Anterior Mediastinum.*—The report of this case was first read at a meeting of the Berlin Medical Society by Dr. Küster. The growth occurred in a healthy-looking, robust man, aged thirty years. He denied syphilitic infection, and there was no evidence of the disease. The tumor projected forward from the right side of the sternum, and adhered to the right border of this bone, involved the third and fourth costal cartilages, and dipped into the chest between them. Negative results followed the administration of iodide of potassium, and the tumor was diagnosed to be sarcoma. In detaching the deeper part, the internal mammary artery was cut, a small aperture was made in the pleural sac, and the lung was seen to collapse immediately. It had also to be detached from the pericardium, under proper antiseptic dressings. The patient made a good recovery, without lung complication. This patient was operated upon Feby. 26, 1882, and exhibited to the Berlin Medical Society in December of the same year. The tumor was found to be a gumma, and the error in diagnosis was admitted.

König's case of excision of an osteo-chondromatous tumor of the sternum, in which both pleural sacs and the pericardium were opened, and the two internal mammary arteries were divided, was referred to. The patient, a woman aged 36 years, recovered. These cases are triumphs of operative skill, and certainly at no time has surgery been so agressive as at the present.

*Transpatellar Excision of the Knee.*—The patient was a lad 13 years of age, who had articular ostitis of the right knee, and excision was decided on (first reported by Mr. Golding Bird at the Clinical Society of London) in this manner: A transverse cut was made across the middle of the patella, which was sawn in two, the two fragments, with the soft parts, being turned up and down. The excision was then completed as usual. Primary union was obtained.

*The Cure of Abscess about the Neck without Cicatrix or other Deformity.*—Dr. Quinlan, of Dublin, recommends the following method: A thin curved needle is armed with silver wire and passed deeply into the swelling, from above downwards, so as to admit of drainage. The ends of the wire are tied together on the outside of the skin, and the part covered with lint wet in alcohol.

This should remain until the abscess is entirely drained. From first to last, fomentations and poultices are interdicted.

A new method of reducing dislocations of the humerus was described, in which the operator used either of the great trochanters as a fulcrum, and the patient placed as close as possible to the edge of the couch, on his back, while the surgeon rotates his body, and has hold of the patient's arm. It is then swung in its proper place.

*A New Method of Curing Hydrocele.*—Consists in the introduction of a bougie into the sac, after the latter has been punctured and evacuated in the usual manner, and left remaining from one to twenty-four hours, has been successfully tried in two hundred and fifty cases.

*Removal of the Uterus with both Ovaries, for Procidentia*, when the womb extended to the knees, with recovery of the patient, concludes the report. This latter operation is certainly a great one ; was performed by Dr. Owens, colleague of the committee.

In the discussion, Dr. G. C. Paoli recited a case of wound of the heart where the patient lived several days, and then died of a complication. He thought it possible that these cases should not all absolutely prove fatal.

Dr. Owens did not think using a bougie as described in the paper a good method to cure hydrocele. He used tr. iodine (warmed) by injecting two or three drachms in the sac and leaving it remain there. It is not necessary to give an anæsthetic to do so. However, to make an incision into the sac and introduce a bougie, would cure these cases, but he did not think it the preferable way. Regarding nephrectomy, he endorsed it, in certain cases, which are, however, rare.

Dr. H. A. Johnson offered the following preamble and resolutions, seconded by Dr. Owens, which were unanimously carried and adopted :

WHEREAS, The library collected under the direction of the Surgeon-General of the U. S. A. is of great value, and is accessible to the profession for study and reference, and

WHEREAS, Provision for the care and direct completion of the index catologue is of the greatest importance, therefore

*Resolved*, That Congress be requested to provide a suitable building for the safe keeping of the collection, and to make provision adequate for its maintenance, and for the completion of the catalogue.

*Resolved*, That, in the judgment of this society, the amount for the maintenance of the library alone should not be less than $10,-000 annually.

*Resolved*, That Congress be asked to provide a commodious fire-proof building for this library and the Army Medical Museum.

Dr. Johnson spoke of the accessibility and great value of this library and museum, and the "Index Medicus" to be used, as it will be the largest medical collection in the world, and no charge will be made to physicians to have access to it. Physicians here can obtain any volume through the librarian of our public library by paying expressage to and from Washington. Senator Logan is Chairman of the Committee in the Senate looking to this object.

Dr. Johnson moved that the secretary send printed copies of the resolutions to Senator Logan and the Surgeon-General, or that a committee be appointed by the chair to take charge of the resolutions, and appeal to Congress to urge their adoption.

The latter motion was seconded by Dr. Owens, and carried. Another motion, by Dr. Johnson, was seconded—that Dr. D. W. Graham should be appointed in his place on the committee from this society to act with the committee from the State Microscopical Society to welcome the National Society of Microscopists to this city in August. Drs. Wm. T. Belfield and J. E. Owens were also added to the committee.

The society then adjourned.

